# Fine-grained evaluation of large language models in medicine using non-parametric cognitive diagnostic modeling

**DOI:** 10.1038/s41598-026-36627-7

**Published:** 2026-01-28

**Authors:** Tianpeng Zheng, Jiayi Liu, Shicong Feng, Zhehan Jiang

**Affiliations:** 1 Institute of Medical Education, Health Science Center, Peking University, Haidian District, Beijing, China; 2 School of Public Health, Peking University, Haidian District, Beijing, China; 3 Graduate School of Education, Peking University, Haidian District, Beijing, China; 4 Peking University Health Science Center-Chaoxing Joint Laboratory for Digital and Smart Medical, Peking University, 38 Xueyuan Rd, Haidian District, Beijing, China

**Keywords:** Large language models, Cognitive diagnostic assessment, Evaluation methods, Medical education, Computational biology and bioinformatics, Health care, Mathematics and computing, Medical research

## Abstract

With the rapid advancement of large language models (LLMs), efficiently and accurately evaluating their capabilities is essential for both developers and users. Unfortunately, most benchmarks evaluate the functionality of LLMs using average scores. This approach oversimplifies evaluation by overlooking nuanced performance differences across specific knowledge domains, failing to provide a comprehensive analysis of the models’ strengths and weaknesses. Safe clinical deployment of LLMs requires moving beyond simple accuracy scores to identify specific knowledge gaps. This study introduces an innovative interdisciplinary approach by integrating measurement theory and psychometric modeling into LLM research, bridging artificial intelligence with educational psychology. Based on 2,809 items from the test bank administrated by National Center for Health Professions Education Development, it employs a non-parametric cognitive diagnostic approach based on cognitive diagnostic assessment to evaluate 41 LLMs performance across 22 medical subdomains. The number of attributes mastered by the evaluated LLMs ranges from 17 to 20. Models with similar total scores can differ notably in their mastery of specific areas, highlighting strengths in some fields and gaps in others. Furthermore, factors such as model size does not always predict comprehensive medical knowledge. The LLMs demonstrate exceptional performance in several areas, achieving 100% mastery in 15 fields such as Cardiology, Dermatology, and Endocrinology etc., underscoring their strong medical knowledge. However, notable variations exist across certain domains. For instance, while Pharmacology and Neuroscience achieve high mastery proportions of 97.56%, Anesthetics & ITU and Emergency Medicine achieve lower proportions of 95.12%. Similarly, radiology has a mastery proportion of 87.80%, while ECG & hypertension & lipids and Liver Disorders show 0%, revealing substantial gaps in these specialized fields. This psychometrically-grounded approach provides multidimensional evaluation of LLMs, identifying specific competency gaps critical for clinical deployment. This methodology serves as an essential quality assurance tool for hospitals, developers, and regulators, enabling domain-specific validation to mitigate risks and ensure patient safety before clinical implementation.

## Introduction

The integration of Large Language Models (LLMs) into high-stakes clinical environments is no longer a future prospect but a rapidly accelerating reality. Recent studies highlight their direct application in critical tasks, such as predicting immunotherapy response in oncology patients^1^, enhancing real-time decision support in anesthesiology^2^, and enabling clinicians to interact with complex ICU data through platforms like ICU-GPT^3^. This swift adoption underscores the immense potential of LLMs to transform healthcare delivery. Compared to other fields, medical decision-making directly impacts patient safety and well-being, necessitating higher standards of accuracy and expertise for LLMs applied in this domain. To rigorously harness the power of LLMs to advance medical practice, it is crucial to accurately evaluate their capabilities in the medical field. Most studies currently evaluate the capabilities of LLMs in the medical field using benchmarks, which offer a standardized evaluation framework, allowing researchers to compare the performance of different models under consistent conditions. Examples of commonly used benchmarks include MedQA dataset^4^, based on the United States Medical Licensing Examination (USMLE); MedMCQA datasets^5^, based on Indian medical entrance exams; and medically focused subsets of the Massive Multitask Language Understanding (MMLU) dataset^6^.

Despite this widespread deployment, the dominant validation paradigm remains dangerously misaligned with the demands of clinical practice. Current benchmarks typically rely on aggregate metrics, such as overall accuracy on medical licensing examinations (e.g., USMLE). While this single-score paradigm offers a convenient summary, it is profoundly limited. It produces an overall accuracy that may look reassuring but often conceals serious vulnerabilities. For example, a model achieving high aggregate performance may still harbor critical knowledge deficiencies in specialized, high-stakes domains such as cardiology—gaps that could have life-threatening implications in real-world use^7^. In effect, this approach fails to capture the model’s competency profile, masking uneven mastery across different areas of medical knowledge and reasoning.

Recognizing this limitation, recent researchers have begun to introduce multi-dimensional benchmarks that evaluate LLMs across subdomains of medical knowledge and cognitive skills. Examples include MultiMedQA^8^, CMB^9^, and MedBench^10^, which provide a more granular view of model performance. These studies have demonstrated that models with comparable overall accuracy can display markedly different strengths and weaknesses. Because interpretability and safety are paramount in medicine, such domain-level differentiation is crucial for identifying where a model is reliable, where it is risky, and how it can be refined or deployed responsibly in clinical workflows. However, even these multi-dimensional benchmarks remain largely grounded in Classical Test Theory (CTT). They typically compute a model’s ability within each subdomain by averaging item-level correctness—treating each item as an equal and independent indicator of the same underlying skill. This assumption rarely holds true in medical testing. In reality, some items simultaneously involve multiple cognitive attributes (e.g., endocrinology and pharmacology), and their difficulties can vary substantially. CTT-based scoring cannot disentangle these intertwined factors, thereby introducing noise and bias into the estimated capability profile.

Obscuring critical performance differences across medical domains and cognitive skills limits the utility of LLMs in real-world applications^7,11^. Without a detailed competency profile, hospital leaders and clinical department heads cannot make evidence-based decisions on which LLM to procure for a specific service line (e.g., oncology vs. cardiology), how to design safe human-in-the-loop workflows, or where to mandate expert oversight. Consequently, any deployment risks being both clinically inappropriate and dangerously unpredictable. Therefore, there is a clear need for a more refined evaluation approach that can dissect and illuminate the cognitive and knowledge-based strengths and weaknesses of LLMs in medicine.

### Cognitive diagnostic assessment: a fine-grained approach

Traditional benchmark evaluations rooted in CTT assume that all items within a given subdomain measure the same latent ability and contribute equally to the total score. This assumption oversimplifies the multidimensional nature of medical reasoning. In contrast, Cognitive Diagnostic Assessment (CDA) offers a fundamentally different perspective. CDA posits that each test item depends on one or more specific skills, such as knowledge units, cognitive processes, or problem-solving strategies, and that an individual’s probability of answering correctly is determined by their mastery of these underlying skills. By inferring examinees’ mastery or non-mastery of each skill from their observed response patterns, CDA enables a fine-grained mapping of their knowledge state (KS)^12^. For instance, a medical test includes three items measuring two attributes: ECG interpretation (A) and drug treatment (B). Student L answers the single-attribute items correctly but fails the combined A + B item, while Student W answers only the combined item correctly. Under CTT, both obtain the same average score, suggesting equal ability. In contrast, CDA identifies that Student L has mastered A and B but struggles with integration, whereas Student W has not mastered either. Therefore, compared with CTT, CDA yields much higher diagnostic resolution, making it especially suitable for answering not just “*how well did the model perform?*” but “*which specific competencies has the model mastered—or failed to master?*”

CDA are widely applied across diverse fields, including psychology, education, and psychopathology^13–15^. In CDA, data analysis methods are typically categorized into two approaches—parametric and non-parametric. Parametric diagnostics rely on psychometric models, such as cognitive diagnostic models (CDMs), to define the theoretical relationship between observed response patterns (ORPs) and KS. Parametric diagnostics are ideal for large-scale assessments^16^, and able to provide detailed parameter insights, including item difficulty and examinees’ higher-order ability levels^12,17,18^. In contrast, non-parametric diagnostic methods, like distance discrimination, classify examinees into latent categories by directly minimizing the distance between ORPs and ideal response patterns (IRPs). Over the past decades, various non-parametric diagnostic methods have been proposed to quantify related assessments, for example, distance discrimination method^16,19^, clustering method^20,21^. Non-parametric approaches, while lacking parametric details, offer distinct advantages that parametric models cannot replicate. First, they simplify analysis by eliminating the need for complex parameter estimation, making the process more efficient and straightforward^20^. Second, they are highly flexible regarding sample size, enabling their application even with minimal datasets, including single observations^19^. Among these methods, weighted general nonparametric classification method (WGNPC) is a saturated method that can be simply converted to other restricted methods, and therefore, has been adopted and investigated more frequently in recent years due to its flexibility and popularity^16,22,23^. In this study, we use WGNPC method through the analysis.

### Current study

This study employs non-parametric cognitive diagnostic methods to evaluate the performance of LLMs across various medical domains, rooted in CDA. The innovation lies in the use of measurement theory and psychometric modeling to LLM studies, leading to an interdisciplinary approach that bridges artificial intelligence with educational and psychological sciences. This interdisciplinary approach moves beyond simple performance metrics to create a granular competency profile for each LLM. Instead of just measuring accuracy, it diagnoses the mastery of specific clinical knowledge domains. This diagnostic capability is essential for identifying high-risk areas before clinical deployment, thereby providing a foundational methodology for the safe and responsible integration of LLMs into healthcare.

## Overview of the nonparametric classification method

### Fundamental concepts

#### Item specification and Q-matrix

CDA provides the foundational framework for understanding how attributes are measured through test items. It ensures that the assessment process aligns with theoretical constructs and accurately evaluates the intended competencies or skills. In the test, item specification defines the attributes that each test item aims to measure. Building on this theoretical foundation, a *Q*-matrix specifies the attributes measured by each item and should accurately represent the theoretical framework of the measurement^24^. Its function is analogous to the assumed factor structure in confirmatory factor analysis^13^. A *Q*-matrix is typically structured as a $\:I\times\:K$ binary matrix, where $\:I$ represents the number of items and $\:K$ represents the number of attributes. In this matrix, $\:{Q}_{ik}=1$indicates that item *i* measures attribute *k*, while $\:{Q}_{ik}=0$ signifies that item *i* does not measure attribute *k*. In a *Q*-matrix, some *q*-vectors may have multiple attributes set to 1, indicating that a single item can simultaneously assess multiple attributes. However, all-zero *q*-vectors are not present, as every item must measure at least one attribute. Table 1 illustrates an example of a *Q*-matrix, offering a clear visualization of the attributes assessed by each item. For example, Item 1 specifically evaluates attribute Neurology, while Item 3 evaluates both attributes Neurology and Rheumatology simultaneously.

**Table 1:** Example of a *Q*-matrix^a^.

Item ID	Neurology	Endocrinology	Rheumatology
1	1	0	0
2	0	1	0
3	1	0	1

^a^1 = the attribute was measured by the task; 0 = the attribute was not measured by the task.

#### ORP and IRP

ORP refers to the actual responses of examinees on a test, typically represented as a binary score: 0 for an incorrect answer and 1 for a correct answer. With $\:K$ attributes, there are $\:{2}^{K}$ possible KS. The IRP corresponds to the expected response pattern for each KS. For example, consider a test with two items, where the *q*-vectors are [1,0] and [1,1], respectively. This test examines two attributes, resulting in four possible KS: [0,0], [1,0], [0,1] and [0,0]. Here, 0 represents a lack of mastery, and 1 represents mastery of an attribute. Assuming an examinee can only answer a question correctly if they have mastered all the attributes required by the item, the IRPs for these four KS are [0,0], [1,0], [0,0] and [1,1], respectively.

Mathematically, the distance discrimination method in non-parametric diagnostic methods generally assigns respondents directly to a latent category by minimizing the distance between the ORP and the IRP.

### Weighted general nonparametric classification method

The Non-Parametric Classification method^19^ is the foundation of WGNPC method. In NPC, with a *I*-item test measuring *K* attributes, the difference between the ORP and the IRP for dichotomous scoring can be quantified using Hamming distance^19^:

1 $$\:{d}_{h}\left({\boldsymbol{y}}_{n},{\boldsymbol{\eta\:}}_{n}\right)={\sum\:}_{i=1}^{I}\left|{y}_{ni}-{\eta\:}_{ni}\right|$$

$\:{\boldsymbol{y}}_{n}=\left({y}_{n1},{y}_{n2},\cdots\:,{y}_{nI}\right)$ represents the ORP of examinee $\:n$, $\:{\boldsymbol{\eta\:}}_{n}=\left({\eta\:}_{n1},{\eta\:}_{n2},\cdots\:,{\eta\:}_{nI}\right)$ denotes the corresponding IRP. $\:{\eta\:}_{ni}={\prod\:}_{k=1}^{K}{\alpha\:}_{nk}^{{q}_{ik}}$, $\:{q}_{ik}=1$ indicates item $\:i\:$measures attribute $\:k$, and $\:{\alpha\:}_{nk}=1$ means examines $\:n$ has mastered attribute $\:k$.

To better address complex linkages between attributes, Chiu et al.^16^ proposed the GNPC method:

2 $$\:{\eta\:}_{li}^{\left(\omega\:\right)}={\omega\:}_{li}{\eta\:}_{li}^{\left(c\right)}+\left(1-{\omega\:}_{li}\right){\eta\:}_{li}^{\left(d\right)}$$

Here, $\:l$ represents the category of KS, with *l* = 1, 2, …, *L*, *L* = 2^*K*^, $\:{\eta\:}_{li}^{\left(\omega\:\right)}$ is the generalized ideal response for examinees in category on $\:l$ item $\:i$. $\:{\eta\:}_{li}^{\left(c\right)}$ and $\:{\eta\:}_{li}^{\left(d\right)}$ correspond to ideal responses under connected and disconnected mechanisms, respectively. $\:{\omega\:}_{li}$ represents the weight reflecting the similarity between $\:{\eta\:}_{li}^{\left(\omega\:\right)}$ and $\:{\eta\:}_{li}^{\left(c\right)}$. $\:{\omega\:}_{li}$ is determined by minimizing the residual distance, given by the formula:

3 $$\:{d}_{li}={{\sum\:}_{n\in\:{C}_{l}}\left({y}_{ni}-{\eta\:}_{li}^{\left(\omega\:\right)}\right)}^{2}$$

The weight formula is:

4 $$\:{\stackrel{\wedge\:}{\omega\:}}_{li}=\frac{{\sum\:}_{n\in\:{C}_{l}}\left({y}_{ni}-{\eta\:}_{li}^{\left(d\right)}\right)}{\lVert{C}_{l}\rVert\left({\eta\:}_{li}^{\left(c\right)}-{\eta\:}_{li}^{\left(d\right)}\right)}$$

$\:{y}_{ni}$ denotes the actual response of examinee $\:n$ on item $\:i$. $\:{C}_{l}$ represents the set of examinees classified into KS category $\:l$, $\:\lVert{C}_{l}\rVert$ indicates the number of examinees in this set.

By incorporating item response variability into the GNPC framework, the WGNPC method is derived. The weighted distance calculation is shown in Eq. (5):

5 $$\:{d}_{w}\left({\boldsymbol{y}}_{n},{\stackrel{\wedge\:}{\boldsymbol{\eta\:}}}_{n}^{\left(\omega\:\right)}\right)={{\sum\:}_{i=1}^{I}\frac{1}{\overline{{p}_{i}}(1-\overline{{p}_{i}})}\left({y}_{ni}-{\stackrel{\wedge\:}{\eta\:}}_{li}^{\left(\omega\:\right)}\right)}^{2}$$

$\:\overline{{p}_{i}}$ represents the passing rate of item $\:i$. When $\:\overline{{p}_{i}}$ = 1 or 0, it is fixed at 0.0001.

## Method

### Assessment dataset

To mitigate the risk of frequently used benchmarks being included in the training data of LLMs, we constructed a novel dataset specifically designed to evaluate the medical knowledge of LLMs. The dataset was sourced from the test bank administrated by the National Center for Health Professions Education Development, which includes 2856 multiple-choice questions (MCQs) spanning 36 subdomains. These items, which contain fictional patient information and clinical scenarios, were developed to test students at the level of pre-licensing (i.e., the end of fourth year of academic programs in China). Importantly, all items in this test bank were expert-reviewed and aligned with the national standards of the physician licensing examination, ensuring high content validity and construct validity. The specialty labels (i.e., the subdomains or attributes each question examines) were also annotated by domain experts, guaranteeing that each item’s cognitive dimension accurately reflects its intended clinical focus.

To ensure the accuracy and stability of the results analysis, attributes and corresponding questions with less than 3 examination times were excluded. After exclusion, a total of 2809 items were retained, involving 22 different subject knowledge points, such as Endocrinology, Respiratory Medicine, Cardiology and so on. Each question examines at least 1 subdomain, and at most 3 subdomains. Based on the subdomains examined by each question, construct the *Q*-matrix. The distribution of questions across these subdomains is presented in Table 2. Each question consists of pure text and includes five answer options, with only one being correct.

### Ethics declaration

The datasets analyzed during the current study are not publicly available due to privacy and confidentiality restrictions involving proprietary test bank items provided by the National Center for Health Professions Education Development. However, the data are available from the corresponding author upon reasonable request and with permission from the data provider. This study did not involve human participants, animals, or patient data. The analyses were conducted using LLMs as test subjects, and therefore ethics approval was not required.

**Table 2:** Distribution of questions across medical subdomains.

Subdomain	Number of questions	Subdomain	Number of questions
Endocrinology	454	Obs & gynae	61
Respiratory medicine	330	Emergency medicine	52
Cardiology	304	ECG & hypertension & lipids	48
Infectious diseases	238	Microbiology	44
Gastroenterology	227	Genitourinary	23
Nephrology	211	Anaesthetics & ITU	21
Haematology	203	Oncology	21
Neurology	203	Liver disorders	15
Rheumatology	201	Pharmacology	6
Miscellaneous	95	Neuroscience	3
Dermatology	93	Radiology	3

### LLM model testing

To ensure robust generalizability of our findings, we evaluated the medical knowledge of 41 popular LLMs, encompassing both open-source models and commercial closed models, as of October 2025. This included both proprietary API-based models (e.g., OpenAI-o1, Gemini-1.5-Pro, and Gemini-2.5-Pro) and open-source models (e.g., Qwen series and MedGemma), spanning a wide range of scales (from 3B to over 100B parameters) and specializations (general-purpose and medical-domain).

**Fig. 1:**
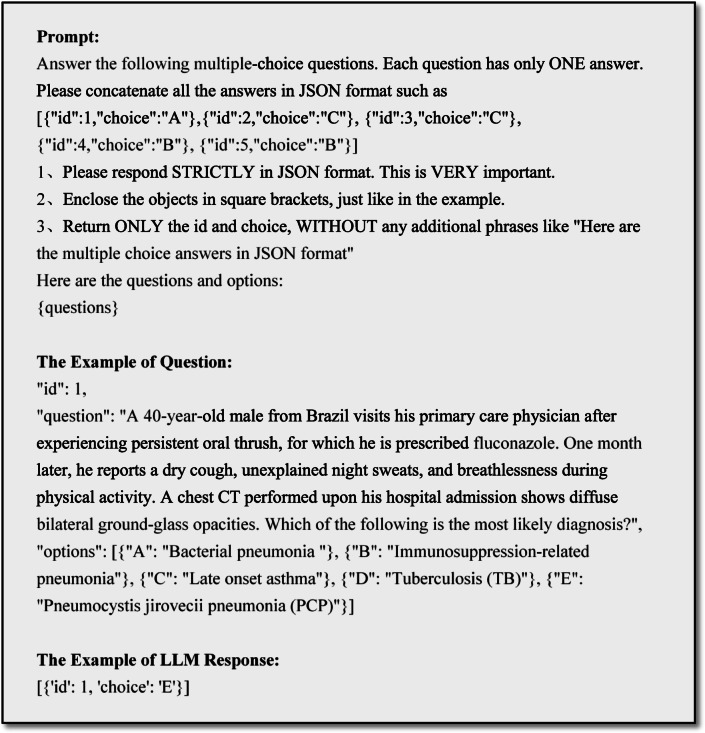
Example of prompt, question, and LLM response.

The evaluation process was implemented via a custom OpenAI-compatible API, leveraging the GPT-based endpoint hosted at an API integration platform^25^. For generation, a temperature of 0.0 was set to ensure deterministic outputs, while a top-p of 1.0 allowed sampling from the full probability distribution of possible continuations. The models were instructed to provide responses in JSON format, facilitating storage and retrieval in a local database for subsequent analysis. Given the substantial influence of prompt engineering on the output of generative LLMs, we standardized the input format of the datasets. The prompt and an example of questions and corresponding LLM responses is illustrated in Fig. 1.

### Data analysis

All analysis was conducted in R software (version 4.4.1). A custom WGNPC code was developed in R to assess the performance of various LLMs on medical examinations. This method was employed to estimate the attribute mastery patterns for each LLM.

## Results

### Reliability and validity

Using classical test theory, the reliability of the entire instrument was assessed with Cronbach’s alpha, yielding a value of α = 0.99. Under CDA framework, both attribute retest consistency and pattern retest consistency were 1, indicating highly stable estimation results.

**Fig. 2:**
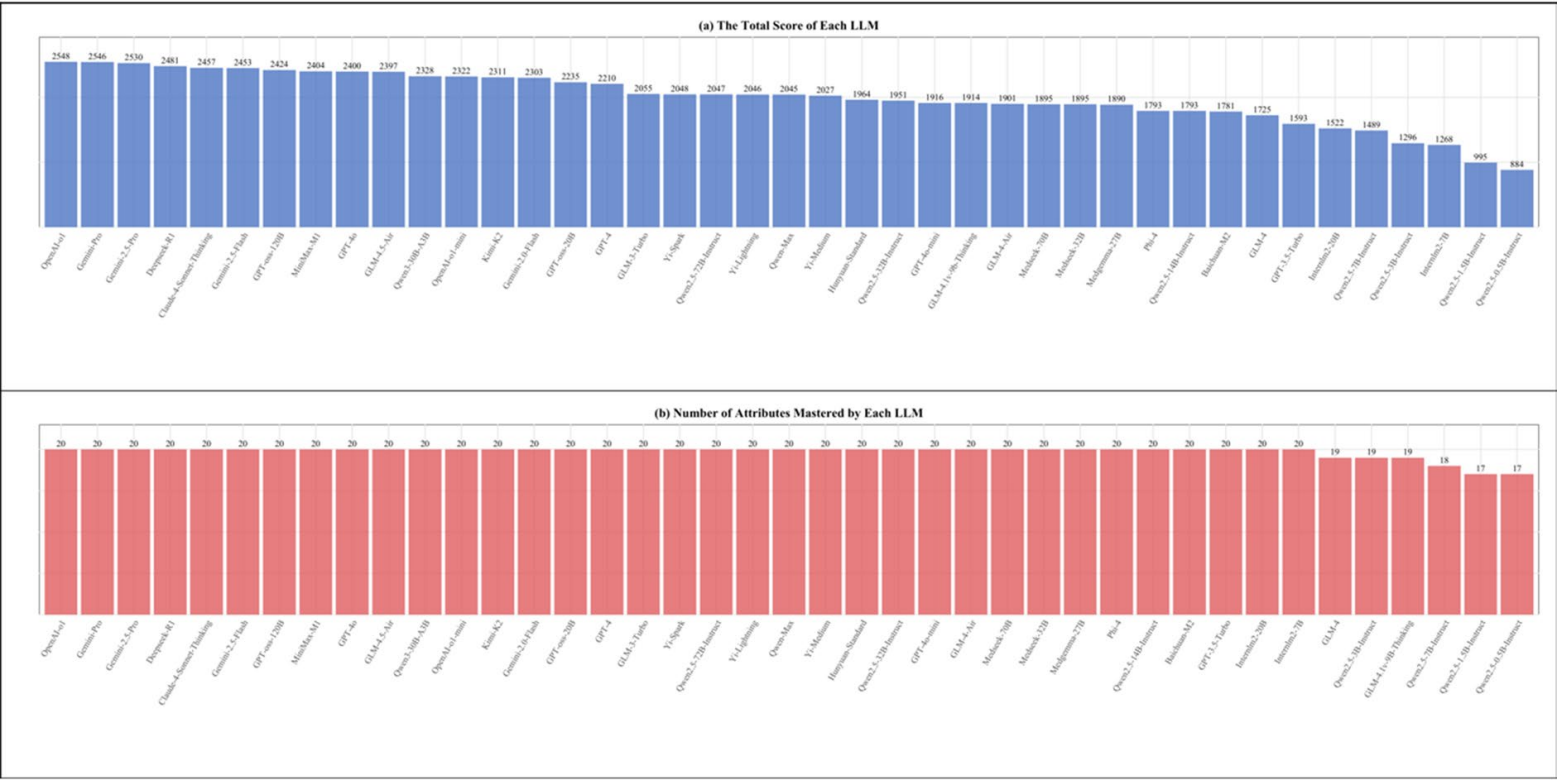
Total scores and number of attributes mastered by each LLM (sorted in descending order).

The performance of various LLMs as presented in Fig. 2a provides a comparative perspective on their capabilities. The total scores, which were calculated based on the number of MCQs correctly answered by each LLM with one point awarded per question. Among the evaluated models, *OpenAI-o1*, *Gemini-1.5-Pro*, and G*emini-2.5-Pro* exhibited the highest overall performance, with total scores of 2548, 2546, and 2530 respectively. In contrast, models such as *Qwen2.5-1.5B-Instruct* and *Qwen2.5-0.5B-Instruct* achieved comparatively lower total scores. The correlation coefficient between overall attribute mastery levels and total test scores was 0.69 (*p* < 0.01), demonstrating well estimation validity^26^. The overall attribute mastery level was calculated by summing the examinee’s KS. For instance, an examinee with a KS of $\:[\mathrm{1,1},\mathrm{1,0},0]$ would have a mastery level of three.

### Diagnostic results

**Fig. 3:**
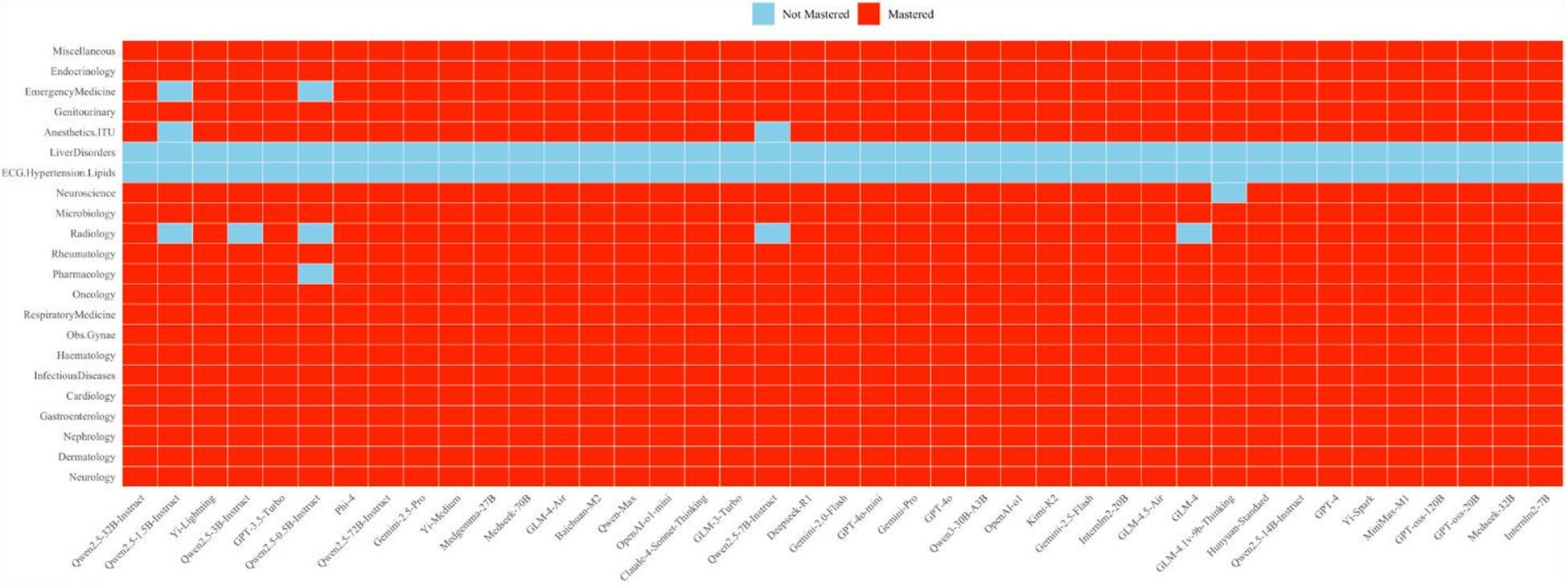
Attribute mastery results for each LLM.

As shown in Figs. 1b, 2 and 3, most LLMs demonstrated broad coverage across medical attributes, with 20 attributes mastered out of the 22 tested. Only six models: *Qwen2.5-3B-Instruct*, *GLM-4*, *GLM-4.1v-9B-Thinking*, *Qwen2.5-7B-Instruct*, *Qwen2.5-1.5B-Instruct* and *Qwen2.5-0.5B-Instruct*, showed worse performance, mastering 17 ~ 19 attributes. Notably, the top-performing models uniquely mastered specialized attributes, such as radiology, which were not achieved by models with fewer mastered attributes. What’s more, some large-sized models (e.g., *OpenAI-o1*, *Gemini-1.5-Pro*, and *Gemini-2.5-Pro*) and medium-sized and small-sized models (e.g., *Medseek-32B*, *GPT-oss-20B*, and *Internlm2-7B*) mastered the same set of attributes, suggesting that a higher parameter size does not always correlate with broader attribute mastery.

**Fig. 4:**
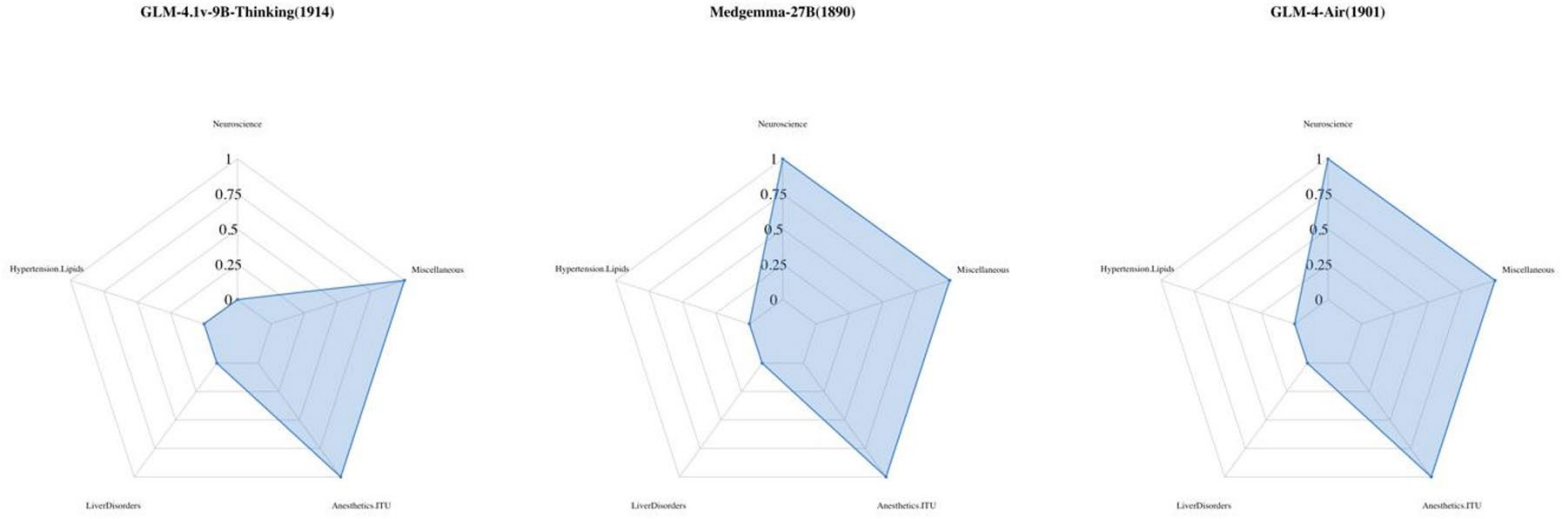
Attributes mastery across three LLMs with similar total scores (the number in parentheses represents the total score of the LLM).

Although some LLMs achieved comparable total scores, their underlying knowledge mastery patterns revealed distinct domain-level differences. For illustrative purposes, Fig. 4 presents the mastery of five assessed attributes by three large language models with similar total scores (*GLM-4.1v-9B-Thinking*: 1914, *Medgemma-27B*: 1890, *GLM-4-Air*: 1901). To enhance clarity, attributes uniformly mastered or not mastered by all models are excluded. The results highlight notable differences in mastery across the five attributes among the models. For example, *GLM-4.1v-9B-Thinking*, despite having the highest raw score among the three, mastered only two attributes, whereas *Medgemma-27B* and *GLM-4-Air* each mastered three. This finding underscores that similar aggregate scores can mask meaningful differences in domain specialization. Therefore, evaluating LLMs solely based on total performance may overlook important variations in their clinical reasoning profiles and domain-specific knowledge representation.

### Distribution of different KS values

**Fig. 5:**
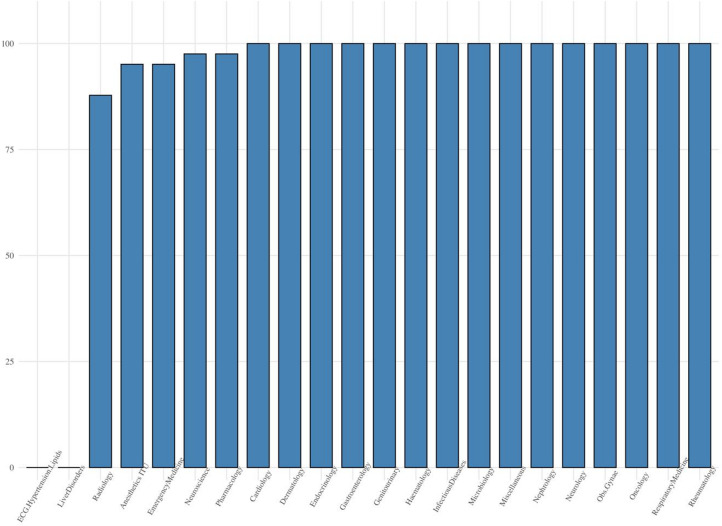
Mastery proportion of each attribute.

Figure 5 depicts the mastery proportions of LLMs across various medical knowledge domains. The LLMs demonstrate exceptional performance in several areas, achieving 100% mastery in 15 fields such as Cardiology, Dermatology, and Endocrinology etc., underscoring their strong medical knowledge. However, notable variations exist across certain domains. For instance, while Pharmacology and Neuroscience achieve high mastery proportions of 97.56%, Radiology is significantly lower at 87.80%. Importantly, ECG & hypertension & lipids and Liver Disorders showed 0%, revealing substantial gaps in these specialized fields.

## Discussion

Previous studies have predominantly evaluated the performance of LLMs using aggregate accuracy scores on standardized benchmarks^4,8,27^. However, relying solely on simple total scores to evaluate the capabilities of LLMs without incorporating measurement theory and psychometric modeling fails to accurately capture their nuanced abilities. In tests, individual items often assess multiple knowledge points. Calculating total scores alone overlooks this complexity, making it challenging to identify and differentiate LLMs’ performance across diverse knowledge areas. While simple scores offer a superficial performance snapshot, they are fundamentally inadequate for clinical risk assessment. A high overall score can create a false sense of security, masking catastrophic deficiencies in high-stakes domains, as demonstrated by our results in areas like ECG & hypertension & lipids and Liver Disorders. This study conducted a comprehensive evaluation of the cognitive performance of multiple LLMs in the medical domain using a non-parametric cognitive diagnostic framework. It provides solutions to questions such as: while both *Medseek-32B* and *GPT-oss-120B* score 90 on MedQA, do they perform equally well in each subarea? The analysis compared performance differences across specific medical domains and highlighted the potential and advantages of this framework in evaluating LLMs’ capabilities in a detailed and multidimensional manner. Instead of a single, ambiguous number, our approach generates a granular clinical competency profile for each LLM. This profile does not just rank models, it provides actionable, diagnostic insights into specific knowledge gaps. This level of detail is not merely a theoretical advancement, it is a prerequisite for any safe and responsible deployment of LLMs in patient care, enabling targeted validation, risk mitigation, and informed decision-making.

The results reveal that high-performing models, such as *OpenAI-o1*, *Gemini-1.5-Pro*, and G*emini-2.5-Pro*, achieved “mastery” across most medical domains attributes, demonstrating a comprehensive understanding of medical concepts. Some models, including *Qwen2.5-3B-Instruct*,* GLM-4*, *GLM-4.1v-9B-Thinking* and *Qwen2.5-7b-Instruct* performed well in general medical domains but showed weaknesses in specialized and complex areas, such as Radiology and Neuroscience. Lower-performing models, such as *Qwen2.5-1.5B-Instruct* and *Qwen2.5-0.5B-Instruct*, mastered only a limited number of medical knowledge attributes. No model mastered ECG & hypertension & lipids and Liver Disorders. These discrepancies highlight areas for improvement, particularly in domains such as ECG & hypertension & lipids and Liver Disorders. This has profound implications for clinical implementation. For instance, a hospital deploying a model like *Deepseek-R1* for Endocrinology might find it useful, but our results serve as a stark warning against its use in the Liver Disorder department. The low mastery in these areas may reflect insufficient training data or the inherent complexity of specific knowledge points. Future efforts to optimize model training should prioritize these specialized fields to enhance models’ comprehensiveness and applicability in medical contexts. Interestingly, the findings indicate that model size and cognitive performance are not strictly positively correlated. While increasing model parameters can enhance performance to some extent^8,28^, targeted optimization of model structure and training data remains crucial. Compared to conventional evaluation methods that rely on aggregate accuracy scores, the non-parametric cognitive diagnostic approach provides a more nuanced analysis, effectively identifying weaknesses and biases across various knowledge dimensions and cognitive processes. This approach offers valuable insights for optimized application deployment. For example, hospitals cannot simply buy the largest model. They require a diagnostic tool like this study to select a model that is truly fit-for-purpose in their specific clinical context. These identified gaps also provide a clear roadmap for AI developers to target their fine-tuning efforts.

### Clinical and practical implications

This study also offers valuable insights into selecting suitable LLMs in clinical practice. First, this framework provides crucial decision support for healthcare organizations. It empowers hospital leaders to move beyond generalized performance claims and select models specifically validated for their intended clinical context, such as ensuring high mastery in oncology for a cancer center. Second, these competency profiles are essential for safety-aware system design. By identifying an LLM’s specific weaknesses (e.g., ECG interpretation), clinical workflows can be engineered to mandate human expert review for high-risk outputs, creating a vital safety net. Finally, our methodology contributes to the broader AI healthcare ecosystem. It offers a clear roadmap for developers to perform targeted model improvements and provides a more robust validation blueprint for regulatory bodies overseeing the approval of clinical AI. Ultimately, our approach serves as a foundational tool for transforming LLMs from promising technologies into trusted and validated clinical assets.

### Limitations

This study has some limitations. First, constrained by the available item labels, the current assessment measured only 22 attributes, all of which represent subject-specific domains. Future research could expand both the dataset and the dimensionality of attributes to include more skill- and cognition-oriented competencies, such as history taking, differential diagnosis, and clinical reasoning, to provide a more comprehensive evaluation of LLM performance. Second, because the primary objective of this study was to establish a fine-grained measurement framework for evaluating LLMs, we adopted multiple-choice questions that provide an objective and standardized basis for comparison. This design, however, may limit the ecological validity of the findings, as LLMs in real clinical contexts are typically confronted with open-ended and complex problem-solving tasks. Therefore, the present study primarily reflects models’ foundational medical knowledge rather than their higher-order reasoning or evidence-based decision-making abilities. Future studies should further explore LLMs’ performance in more complex, context-rich clinical scenarios to better capture their reasoning and decision-making capacities. Third, a primary limitation of the current study is the reliance on a single, standardized zero-shot prompt format. While this ensured consistency for inter-model comparisons using the CDA framework, the diagnostic profiles may exhibit sensitivity to prompt variations (e.g., few-shot prompting and chain-of-thought instructions). Future research must include systematic ablation studies on prompt effects to rigorously test the stability of CDA-derived attribute mastery profiles. Fourth, while the WGNPC method employed in this study incorporates statistical weighting based on item variance to enhance classification accuracy, there remains room for methodological refinement in future evaluations. The current weighting relies solely on psychometric properties derived from response data. However, in the context of medical AI safety, not all errors carry equal consequences. Future research could explore clinically-oriented weighting strategies, where items involving life-threatening scenarios or high-stakes clinical decisions are assigned higher weights by domain experts. Lastly, given the rapid advancements in LLMs and the increasing integration with Internet-based functionalities, the performance of these models is continually evolving. Future studies could consider conducting follow-up evaluations at regular intervals to capture the evolving capabilities of LLMs. Nevertheless, this study still offers a valuable framework for assessing both open-source and commercial LLMs’ performance. By addressing these limitations and building on the current findings, future research can further enhance our understanding of LLM performance in medical contexts and contribute to the development of more robust and reliable models for healthcare applications.

## Conclusion

In conclusion, this study conducted a critical insight for the era of AI in medicine: overall performance scores mask potentially catastrophic weaknesses in LLMs. By uncovering profound knowledge gaps in high-stakes specialties like oncology and emergency medicine, our work demonstrates that a psychometrically-grounded assessment is not merely a better evaluation method, but an essential prerequisite for safe clinical deployment. This framework provides an indispensable toolkit for hospital leaders, clinicians, and developers to mitigate risks, make informed implementation decisions, and ultimately ensure that AI integration into patient care is both responsible and trustworthy.

## Data Availability

The datasets analyzed during the current study are not publicly available due to privacy and confidentiality concerns related to the proprietary test bank items from the National Center for Health Professions Education Development but are available from the corresponding author on reasonable request.

## Abbreviations

LLMs: Large language models
CDA: Cognitive diagnostic assessment
KS: Knowledge state
CDMs: Cognitive diagnostic models
ORPs: Observed response patterns
IRPs: Ideal response patterns
WGNPC: Weighted general nonparametric classification method
MCQs: Multiple-choice questions
